# Supplementary material for: Bioactivity assessment of natural compounds using machine learning models trained on target similarity between drugs
Source: PLoS Comput Biol. 2022 Apr 25;18(4):e1010029. doi: 10.1371/journal.pcbi.1010029 (PMC9071136; doi:10.1371/journal.pcbi.1010029)
Supplement: S3 Table — (DOCX) [file pcbi.1010029.s003.docx]

**S3 Table** Model performance metrics and their description: TP – true positive, FP – false positive, TN – true negative, FN – false negative, TPR – true positive rate (TP/Positives), TNR – true negative rate (TN/negatives)

| **Performance metric** | **Formula** |
| --- | --- |
| Matthews correlation coefficient (MCC) | $MCC= \frac{TPXTN-FPXFN}{\sqrt{\left( TP+FP \right)\left( TP+FN \right)(TN+FP)(TN+FN)}}$ |
| F1 score | $F1= \frac{2TP}{2TP+FP+FN}$ |
| Balanced accuracy (BAC) | $BAC= \frac{TPR+TNR}{2}$ |
| Kappa statistic | $K=\frac{2X(TPXTN-FNXFP)}{\left( TP+FP \right)X\left( FP+TN \right)+\left( TP+FN \right)X(FN+TN)}$ |
| Positive predictive value (PPV) | $PPV= \frac{TP}{TP+FP}$ |
| Accuracy | $ACC= \frac{TP+TN}{Positives+Negatives}$ |
| Mean misclassification error (MMCE) | Defined as: mean(response != truth) |
