# Supplementary material for: Bioactivity assessment of natural compounds using machine learning models trained on target similarity between drugs
Source: PLoS Comput Biol. 2022 Apr 25;18(4):e1010029. doi: 10.1371/journal.pcbi.1010029 (PMC9071136; doi:10.1371/journal.pcbi.1010029)
Supplement: S5 Table — Relative Inhibition (%) (DOCX) [file pcbi.1010029.s005.docx]

**S5 Table** Cox-assay results. Relative Inhibition (%)

| Conc.(μM) | Conc. (log) | Triflusal | 5-Methoxysalicylic acid | 4-Isopropylbenzoic acid |
| --- | --- | --- | --- | --- |
| 400 | 2.60205999 | 50.2711928 | 44.5041162 | -106.98147 |
| 200 | 2.30103 | 53.7473155 | 41.4790048 | -38.982039 |
| 100 | 2 | 49.9543006 | 40.2151692 | -0.4554861 |
| 50 | 1.69897 | 44.7753213 | 36.7105663 | 10.6613523 |
| 25 | 1.39794001 | 41.6782677 | 32.5953934 | 14.4918464 |
| 12.5 | 1.09691001 | 41.3314119 | 33.0239321 | 17.6503106 |
