# Supplementary figures and images for: Bioactivity assessment of natural compounds using machine learning models trained on target similarity between drugs
Source: PLoS Comput Biol. 2022 Apr 25;18(4):e1010029. doi: 10.1371/journal.pcbi.1010029 (PMC9071136; doi:10.1371/journal.pcbi.1010029)

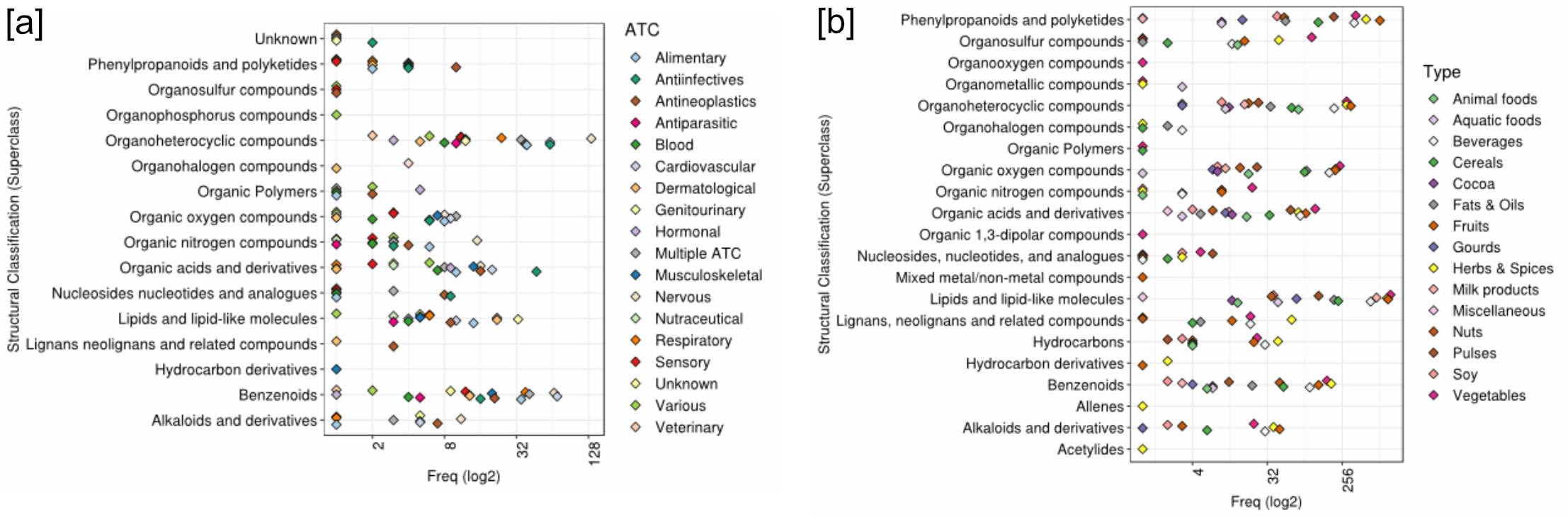

Supplement: S1 Fig — (TIFF) [file pcbi.1010029.s006.tiff]

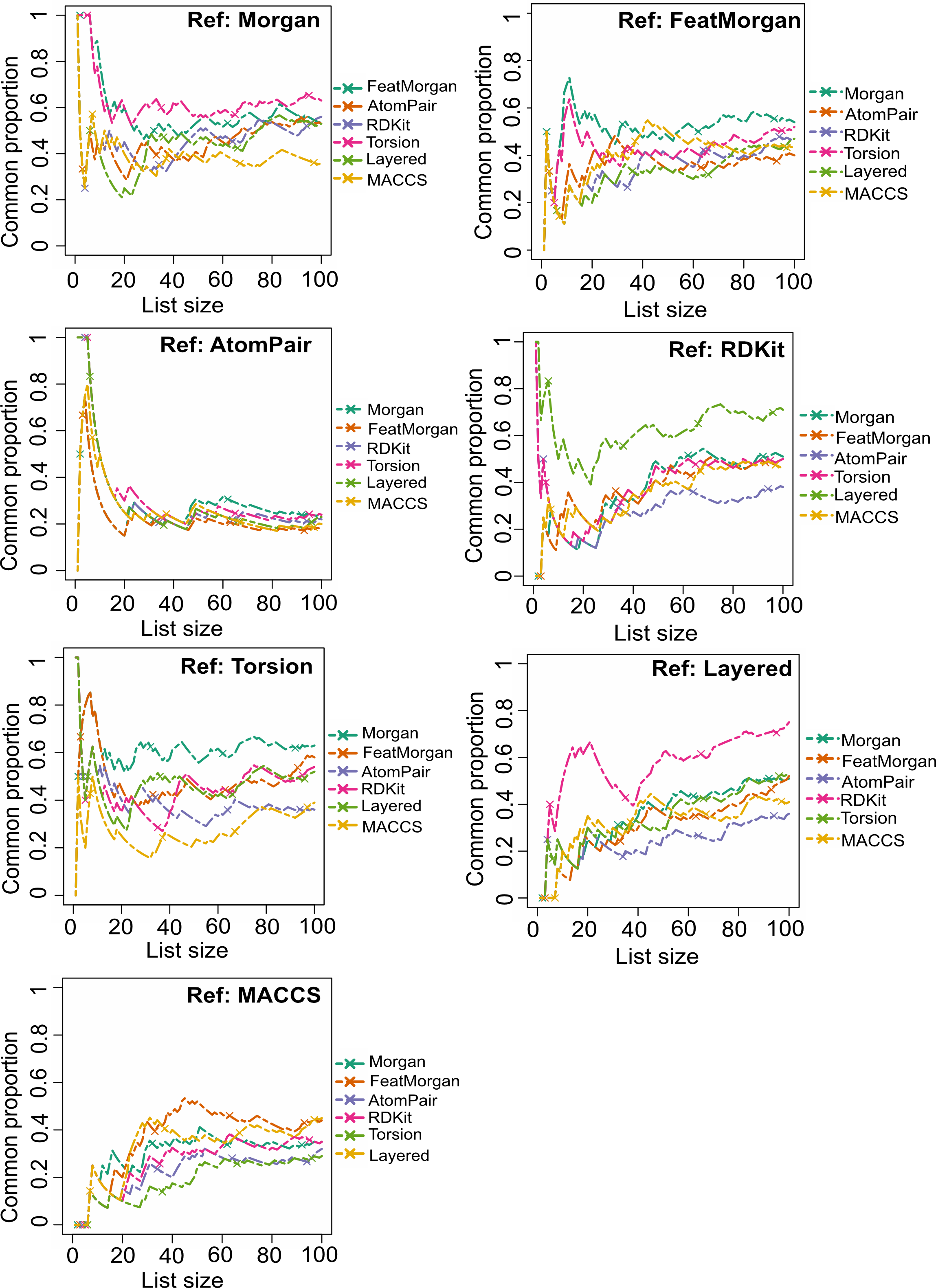

Supplement: S2 Fig — ‘Concordance at top’ (CAT plots)55 of fingerprints. We performed a rank-based assessment of drug-pairs called by each fingerprint as top scoring pairs and found a low concordance between them. The CAT plots here depict the concordance of each fingerprint (when taken as reference) against all other fingerprints. Each fingerprint was taken as a reference and the top 100 high scoring drug-pairs were ranked in decreasing order to estimate the overlapping proportions between each fingerprint when compared with the reference. Mathematically, for ith top-ranked molecules, concordance is defined as length(intersect(list1[1:i],list2[1:i]))/i. (TIFF) [file pcbi.1010029.s007.tiff]

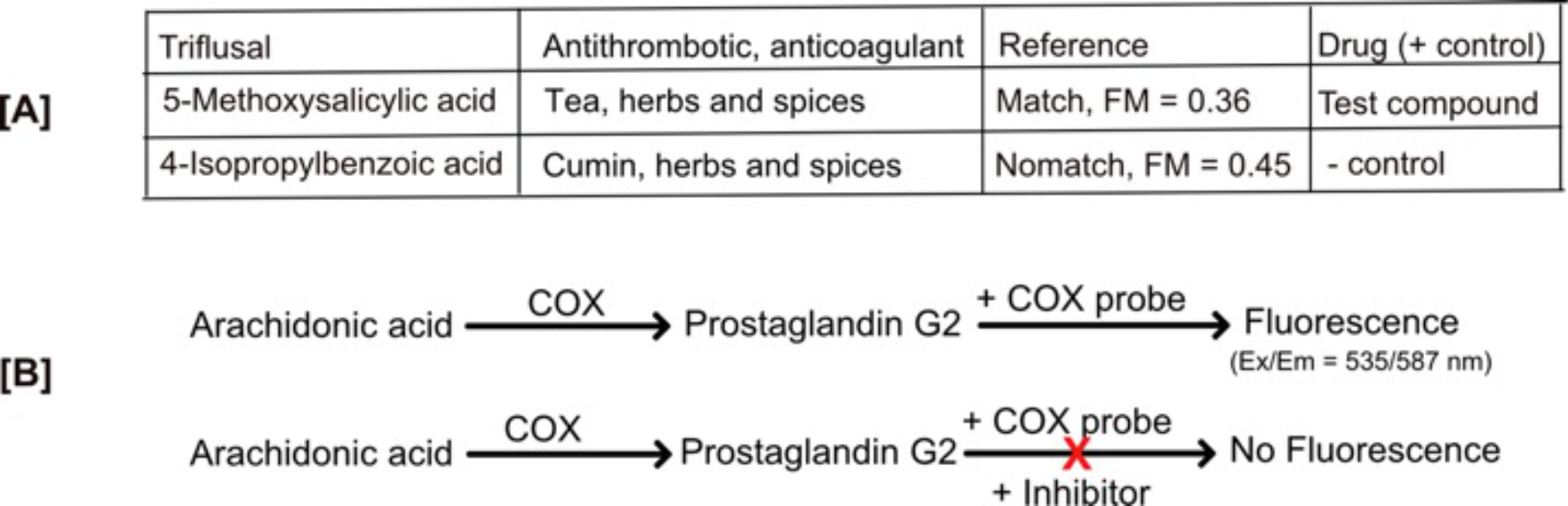

Supplement: S3 Fig — (A) Table describes the compounds tested in the cox-1 inhibitor assay, their source and usage. Triflusal is the drug which is known to bind to Cox-1 and it was a positive control in our experiment. The food compound which came out as hit with our prediction models is 5-methoxysalicylic acid (referred to as test compound) for which target engagement is being studied. Additional inclusion was 4-isopropylbenzoic acid (selected based on high FM score as compared to test compound but deemed no match by prediction models) as a negative control. (B) The reaction mechanism involves fluorometric detection of intermediate product (prostaglandin G2) generated by cox-enzyme. (TIFF) [file pcbi.1010029.s008.tiff]
